# Critical Low Catastrophe: A Case Report of Treatment-Refractory Hypoglycemia following Overdose of Long-Acting Insulin

**DOI:** 10.1155/2020/8856022

**Published:** 2020-10-19

**Authors:** Rashi Sandooja, John M. Moorman, Monisha Priyadarshini Kumar, Karla Detoya

**Affiliations:** ^1^Department of Internal Medicine, Cleveland Clinic Akron General, Akron, OH, USA; ^2^Department of Pharmacy Practice, Northeast Ohio Medical University, Rootstown, OH, USA; ^3^Department of Endocrinology, Cleveland Clinic Akron General, Akron, OH, USA

## Abstract

Overdose of long-acting insulin can cause unpredictable hypoglycemia for prolonged periods of time. The initial treatment of hypoglycemia includes oral carbohydrate intake as able and/or parenteral dextrose infusion. Refractory hypoglycemia following these interventions presents a clinical challenge in the absence of clear guidelines for management. Octreotide has sometimes been used, but its use is generally limited to sulfonylurea overdose. In this case report, we present a case of refractory hypoglycemia following an overdose of 900 units of long-acting insulin glargine that failed to respond to usual modes of therapy mentioned above. Stress-dose corticosteroids were then initiated, followed by subsequent improvement in IV dextrose and glucagon requirements and blood glucose levels. Hence, corticosteroids may serve as an adjunctive therapy in managing hypoglycemia and can be considered earlier in the course of treatment in patients with refractory hypoglycemia to prevent volume overload, especially when large volumes of dextrose infusions are required.

## 1. Introduction

Insulin is a mainstay of treatment in patients with type 1 diabetes mellitus (T1DM), and a significant proportion of patients with type 2 diabetes mellitus (T2DM) are insulin-dependent [1–3]. At the same time, insulin therapy is associated with multiple adverse effects, with hypoglycemia being one of the most common. For example, in the U.K. Prospective Diabetes Study, the prevalence of hypoglycemia in patients with T2DM who were taking insulin was 11.2%, as compared to 3.3% in those taking sulfonylureas and 2.4% in those taking metformin [4]. However, these prevalences represent usual medication use and do not account for the potential for insulin overdose, either intentional or unintentional. Management of insulin overdose poses a clinical challenge and may require modified treatment strategies, given the lack of consensus and guidelines. The objective of this report is to describe a case of intentional insulin overdose associated with refractory hypoglycemia and to discuss the integrative approach to managing this case.

## 2. Case Report

A 27-year-old female with a history of bipolar 1 disorder, borderline personality disorder, posttraumatic stress disorder (PTSD), previous history of multiple suicide attempts, T2DM, and obesity (weight 160 kg; body mass index 56 kg/m^2^) status-postsleeve gastrectomy in 2016 presented to the emergency department (ED) following a suicide attempt. She was brought to the ED by self-notifying emergency services after having shortness of breath and dizziness following self-injection of 900 units of insulin glargine U-100 (Lantus®) subcutaneously into her abdomen three hours prior. This represented her third intentional insulin overdose in the preceding six months. She was previously insulin-requiring, on Lantus, but was weaned off insulin four years ago after sleeve gastrectomy and subsequent 68 kg weight loss over the preceding five years. The patient's most recent hemoglobin A1c (HbA1c) ten months prior to presentation, while off insulin or any other hypoglycemic agents, was 4.7%. Home medications at this time included duloxetine 120 mg daily, bupropion 300 mg daily, lithium 900 mg daily, prazosin 5 mg at bedtime, trazodone 150 mg at bedtime, and mirtazapine 30 mg at bedtime.

The patient reported suicidal ideation and worsened depression for three months prior to presentation and decided to inject three full insulin glargine U-100 pens (300 units each), which she had at home from her prior insulin prescription. The initial fingerstick blood glucose taken by paramedics prior to arrival to the ED was 131 mg/dL. At that time, she was closely monitored, but not medically treated, and was sent to the ED. Her fingerstick blood glucose level upon presentation to the ED was 113 mg/dL, at which time she was somnolent but arousable and answering questions appropriately. Her physical examination upon arrival was unremarkable and did not reveal any areas of fluctuance at the reported injection sites.

**Figure 1:**
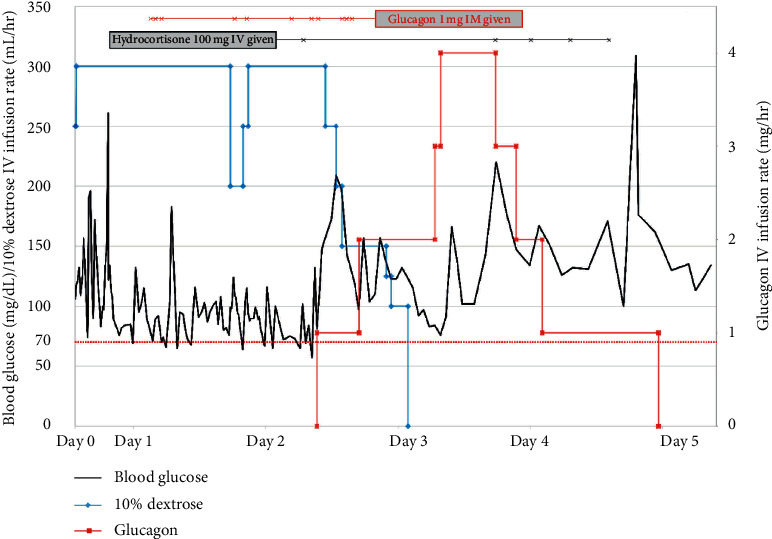
Blood glucose trends over time starting at ED presentation (black line, left axis), along with IV dextrose infusion rate (blue line, left axis), IV glucagon infusion rate (red line, right axis), hydrocortisone IV doses (black horizontal line across the top), and glucagon IM doses (red horizontal line across the top).

Figure 1 depicts the patient's course of therapy from the time of ED presentation. An intravenous (IV) 10% dextrose (D10) infusion was started at 300 mL/hour 30 minutes after ED arrival to prevent hypoglycemia. A regular diet was started and carbohydrate intake was encouraged. Fingerstick glucose levels were monitored every 15 minutes for the first seven hours and every 30 minutes thereafter. The patient's blood glucose readings first approached the hypoglycemic range of <70 mg/dL approximately three hours after presentation (fingerstick blood glucose = 74 mg/dL). She received two 50-gram ampules of dextrose 50% (D50) IV, along with three 1 mg doses of intramuscular (IM) glucagon, over the next four hours in addition to the D10 IV infusion. The patient was then transferred to the medical intensive care unit (MICU) for close observation.

Due to persistent hypoglycemia and inability to wean the D10 IV infusion, a standing order for glucagon 1 mg IM for blood glucose <70 mg/dL was added. During the first 48 hours of her admission, she required eleven 1 mg IM glucagon doses in addition to the D10 IV infusion but continued to experience hypoglycemia with the lowest recorded blood glucose reading of 64 mg/dl. She was transitioned from a standard diet to a bariatric diet due to suspected reactive hypoglycemia associated with her postbariatric surgery status, which consisted of 56 grams of carbohydrate and 18 grams of protein per day. An IV glucagon infusion was started at 1 mg/hr on hospital day two (approximately 44 hours after presentation) in a continued effort to minimize IV fluid volume. In addition, a trial dose of hydrocortisone 100 mg IV bolus was given at the time the IV glucagon infusion was started. The IV glucagon infusion was titrated to 2 mg/hr as the D10 IV infusion was weaned off, which was done on hospital day three (approximately 73 hours after presentation). However, the patient's glucose levels began trending towards the hypoglycemic range, requiring further titration of the IV glucagon infusion to 4 mg/hr. Hydrocortisone 100 mg IV every 8 hours was then started to reduce the IV and IM glucagon requirements. The IV glucagon infusion was weaned off on hospital day four after three additional doses of hydrocortisone on days 3 and 4. The patient became hyperglycemic following the last dose of hydrocortisone (blood glucose = 309 mg/dL), which prompted discontinuation of the stress-dose steroids. Following discontinuation of medical therapy for hypoglycemia, her blood glucose ranged from 130 to 150 mg/dL, and the patient was transferred out of the MICU to a medical floor for observation without recurrence of hypoglycemia.

## 3. Discussion

Patients who experience an insulin overdose can present with prolonged, refractory hypoglycemia, especially with the administration of long-acting insulin [5]. Although the duration of action of insulin glargine is reported to be 24 to 30 hours [6], these parameters were observed with doses of 0.3 to 0.5 units/kg, whereas the patient in the present case self-administered a dose of 5.6 units/kg [7]. In cases of overdose, the duration of action may be affected by a number of factors, including the route and site of administration, injection volume, solution concentration (e.g., U-100 vs. U-300), local blood supply, and presence of lipodystrophy or cutaneous amyloidosis at the injection site. For example, insulin injections of larger volumes are more slowly absorbed from the subcutaneous space than smaller volumes, which may result in delayed hypoglycemia following the injection [8]. Other factors, such as the presence of insulin autoantibodies and renal dysfunction, can prolong the duration of hypoglycemia as well. Therefore, it may be difficult to predict the duration of hypoglycemia in cases of insulin overdose. Based on case reports involving insulin overdose, hypoglycemia has been reported up to several days after injection [5, 9, 10].

In patients who present with hypoglycemia, current clinical practice guidelines suggest a tiered approach to treatment based on the initial blood glucose level and the patient's ability to self-treat [11, 12]. The initial treatment for hypoglycemia should primarily involve oral carbohydrate ingestion [11]. However, if the patient is unable to ingest carbohydrates or if hypoglycemia persists despite this, parenteral dextrose and/or glucagon may be administered [12]. This should be accompanied by frequent monitoring of serum glucose levels and electrolytes, especially serum potassium. Continuous infusions of dextrose (e.g., 5% or 10% solutions) may be necessary depending on the patient's response to initial therapy and the ability to eat food [12].

In cases where hypoglycemia persists despite standard treatment modalities, other treatment options can be explored. The somatostatin analog octreotide has been tried in previous cases of insulin overdose [13, 14]. However, its use was associated with inconsistent results and is usually reserved for hypoglycemia secondary to sulfonylurea use [15]. Another uncommon approach to cases of insulin overdose includes excision of the insulin depot at the injection site when clear fluctuance is present. This theoretically can help in preventing delayed absorption of insulin and associated prolonged hypoglycemia and has been utilized successfully in previous cases [16, 17]. However, in cases such as the present one where an area of fluctuance is not visible on physical examination, this option is not possible.

Another treatment strategy that may be underutilized involves the use of glucocorticoids. These agents promote hepatic gluconeogenesis and decrease peripheral glucose uptake and are frequently associated with hyperglycemia as a result. Hence, glucocorticoids may serve as an adjunctive therapy option in managing refractory hypoglycemia in cases of insulin overdose. This approach has been utilized in a prior case of refractory hypoglycemia associated with insulin overdose where hydrocortisone was administered to prevent volume overload associated with the infusion of large volumes of dextrose-containing IV fluids [14]. Early initiation of steroids in cases with persistent hypoglycemia may reduce the duration of intensive care unit stay, avoid invasive procedures like central line placement for 25% or 50% dextrose infusions, and prevent volume overload from large volume dextrose infusions [18].

This patient presented with intentional insulin overdose after injecting 900 units (∼5.6 units/kg) of insulin glargine U-100 (Lantus®). Her blood glucose levels first approached the hypoglycemic range of <70 mg/dL about six hours after she reported injecting the insulin. The initial management of her hypoglycemic episodes followed guideline recommendations, starting with oral carbohydrate ingestion and parenteral dextrose administration [11, 12]. One concern raised with this approach was regarding her history of sleeve gastrectomy and that the carbohydrate ingestion could lead to excess endogenous insulin release and subsequent worsening of hypoglycemia symptoms [19]. Therefore, the decision was made to place her on a high protein, low carbohydrate bariatric diet to help mitigate this potential issue. Adjunctive measures were required due to her persistent hypoglycemia, and she was therefore given IM glucagon injections in addition to oral and parenteral carbohydrates. An IV glucagon infusion was tried as well, which proved ineffective at completely resolving hypoglycemia.

In the setting of persistent hypoglycemia three days after the reported overdose and concern for volume overload, other treatment modalities were considered. Octreotide was not given due to the inconsistent results from previous cases along with its high cost and because sulfonylurea coingestion was not suspected [13, 14]. Excision of the insulin depot was not possible in our patient because she did not have a clear site of fluctuance at the injection site. Consistent with use of hydrocortisone in a previous case report [14], a trial of stress-dose hydrocortisone was started. Hydrocortisone was chosen due to its shorter duration of action compared to other glucocorticoids, thereby reducing the risk of subsequent hyperglycemia. Following the administration of four doses of hydrocortisone, she was able to be weaned off both the D10 and glucagon infusions and was able to maintain euglycemia.

There are some important limitations to the present case that should be considered as well. We were unable to obtain the insulin product to verify its identity, and there was no recent prescription history to reconcile since the patient was no longer on insulin. Therefore, it is possible that the insulin product administered was not as stated and/or the insulin product was expired. However, given the timeline of events and the intensive medical therapy that she required, it seems reasonable to assume that long-acting insulin with adequate potency was used. We did not rule out other secondary causes of hypoglycemia, such as postprandial hyperinsulinemic hypoglycemia associated with bariatric surgery. However, this is more commonly associated with Roux-en-Y gastric bypass than sleeve gastrectomy and was thought to be more likely associated with the insulin overdose in the present case given the temporal association with insulin administration [19]. Given the prior cases involving the use of glucocorticoids for insulin-induced hypoglycemia, we felt that the administration of hydrocortisone contributed towards the resolution of hypoglycemia and reduced the continued need for parenteral dextrose and glucagon in the present case. However, given the delay in the administration of hydrocortisone until three days after presentation, it is also possible that the hypoglycemia resolved due to the clearance of the exogenous insulin, as opposed to the effect of glucocorticoid administration. Finally, while the efficacy of IV glucagon infusion is more established in pediatrics with T1DM and in neonatal hypoglycemia, the efficacy of continuous infusions of IV glucagon for the treatment of insulin-induced hypoglycemia in adults is unclear given the absence of strong supporting evidence in this population [14, 20, 21]. Glucagon acts by promoting glycogenolysis and gluconeogenesis and thus has limited use once glycogen stores have been depleted. On the other hand, insulin itself promotes the conversion of glucose to glycogen for storage in the liver. Importantly, this was this patient's third suicide attempt with insulin, the first with 300 units of insulin lispro U-100 and the second with 300 units of insulin glargine U-100. These previous overdoses were managed successfully with IV glucagon infusions; therefore, it was reasonable to attempt the use of an IV glucagon infusion for persistent hypoglycemia during the present case. However, her prior suicide attempts involved much smaller doses of insulin, which may explain why the IV glucagon infusion alone failed to resolve hypoglycemia in the present case, thereby requiring hydrocortisone administration.

## 4. Conclusions

Insulin overdose often requires intensive care admission and frequent monitoring of blood glucose and electrolytes. Prolonged continuous D10 IV infusions can lead to volume overload, and parenteral administration of higher concentrations of dextrose (e.g. 25% or 50%) requires invasive procedures to obtain central venous access [18]. This case adds to the literature supporting the use of glucocorticoids in the management of refractory hypoglycemia associated with overdoses of long-acting insulin. Additionally, this case involved a patient who was not being treated for diabetes mellitus following sleeve gastrectomy and could be extrapolated to such patients. Early initiation of hydrocortisone should be considered in cases of refractory hypoglycemia secondary to overdose of long-acting insulin products to prevent unnecessary interventions, decrease IV fluid requirements, and reduce the risks associated with prolonged hypoglycemia.
